# Validity and reliability of the English and translated Chinese versions of the Integrated Palliative care Outcome Scale (IPOS) in Singapore

**DOI:** 10.1186/s12904-021-00737-y

**Published:** 2021-03-09

**Authors:** Victoria Jane En Long, Yin Bun Cheung, Debra Qu, Katherine Lim, Guozhang Lee, Alethea C. P. Yee, Ping Guo, Richard Harding, Grace Meijuan Yang

**Affiliations:** 1 Duke-NUS Medical School, Singapore, Singapore; 2 Tampere University, Tampere, Finland; 3 National Cancer Centre Singapore, Singapore, Singapore; 4 Dover Park Hospice, Singapore, Singapore; 5 Singapore General Hospital, Singapore, Singapore; 6 Assisi Hospice, Singapore, Singapore; 7 School of Nursing, Institute of Clinical Sciences, College of Medical and Dental Sciences, University of Birmingham, Birmingham, UK; 8 Cicely Saunders Institute, Florence Nightingale Faculty of Nursing, Midwifery and Palliative Care, King’s College London, London, UK

**Keywords:** Palliative care, Patient-centred outcomes, Advanced cancer, IPOS, Reliability, Validity

## Abstract

**Context:**

Measurement of patient-centred outcomes enables clinicians to focus on patient and family priorities and enables quality of palliative care to be assessed.

**Objectives:**

This study aimed to evaluate the validity and reliability of the English and translated Chinese versions of the Integrated Palliative care Outcome Scale (IPOS) among advanced cancer patients in Singapore.

**Methods:**

IPOS was forward and backward translated from English into Chinese. Structural validity was assessed by confirmatory factor analysis; known-group validity by comparing inpatients and community patients; construct validity by correlating IPOS with Edmonton Symptom Assessment System-revised (ESAS-r) and Functional Assessment of Cancer Therapy–General (FACT-G); internal consistency by Cronbach’s alpha; inter-rater reliability between patient and staff responses; test-retest reliability of patient responses between two timepoints.

**Results:**

One hundred eleven English-responding and 109 Chinese-responding patients participated. The three-factor structure (Physical Symptoms, Emotional Symptoms and Communication and Practical Issues) was confirmed with Comparative Fit Index and Tucker-Lewis-Index > 0.9 and Root Mean Square Error of Approximation < 0.08. Inpatients scored higher than outpatients as hypothesised. Construct validity (Pearson’s correlation coefficient, r ≥ |0.608|) was shown between the related subscales of IPOS and FACT-G and ESAS-r. Internal consistency was confirmed for total and subscale scores (Cronbach’s alpha≥0.84), except for the Communication and Practical Issues subscale (Cronbach’s alpha = 0.29–0.65). Inter-rater reliability (Intra-class correlation coefficient [ICC] ≤ 0.43) between patient and staff responses was insufficient. Test-retest reliability was confirmed with Intra-class correlation coefficient ICC = 0.80 (English) and 0.88 (Chinese) for IPOS Total.

**Conclusion:**

IPOS in English and Chinese showed good validity, good internal consistency, and good test-retest reliability, except for the Communication and Practical Issues subscale. There was poor inter-rater reliability between patients and staff.

## Key message

This article demonstrates that the Integrated Palliative care Outcome Scale (IPOS) and its translated Chinese version is a valid tool to measure palliative care outcomes in advanced cancer patients in Singapore. However, the staff version is a poor proxy measure, which leaves room to develop an alternative caregiver proxy measure.

## Background

Patient reported outcome measures (PROMs) are patient-centred questionnaires that measure perceived functional status and wellbeing [1]. PROMs can be used to evaluate various healthcare inventions to identify efficient ways of delivering health care [2]. PROMs facilitate clinical practice by promoting patient-centred communication, screening for unmet needs and monitoring the severity of problems related to disease or treatment toxicity [3]. Such patient-centred data is particularly useful in palliative care, improving awareness of unmet need, enabling professionals to act to address patients’ needs, and benefitting patients’ emotional and psychological quality of life [4]. However, palliative care involves comprehensive assessment and management of problems in multiple domains. For patients who are often fatigued from their serious illness, this may result in use of multiple survey instruments that are too lengthy to complete. Therefore, a brief measure is needed for the context of palliative care.

The Integrated Palliative care Outcome Scale (IPOS) is a brief measure of palliative care problems, covering multiple domains of physical and psychological symptoms, social and spiritual issues, communication, information needs and practical concerns [5]. IPOS was adapted from the Palliative care Outcome Scale (POS) [https://pos-pal.org/]. POS is a widely used and validated PROM, being translated into 12 languages and adapted for use in specific clinical populations such as dementia [6]. It has proven to be an important and relevant tool in improving the practice of palliative care, through the assessment of patient and caregiver’s quality of life (QOL), improving healthcare interventions and in the development of other PROMs [7]. Similar to POS, IPOS also has a staff proxy version [5]. This is particularly pertinent in palliative care, where patients often suffer from cognitive impairment and deteriorating health over time which renders them unable to complete questionnaires on their own [6]. IPOS was originally developed in English and has been translated into different languages such as French, Japanese, German and Czech, and has shown good validity across different cultural contexts [5, 8–10].

The main aim of this study was to validate the English and translated Chinese versions of IPOS among advanced cancer patients in Singapore. This validation study complements the growing list of IPOS versions validated in different languages, which potentially paves the way for multinational, cross-cultural studies of palliative care outcomes.

## Methods

### Participants and procedure

#### Setting

Singapore is a multi-ethnic country, with the major ethnic groups being Chinese (74%), Malay (13%) and Indian (9%) [11]. Among the population aged 15 and above in Singapore, 94.7% of the population are literate in English, Chinese or both languages, with 15.6% literate in Chinese only [12].

#### Translation and cultural adaptation

Guidelines for the POS family of measures were used for translation of the Chinese version and cultural adaptation of both patient versions [13]. The procedure for the Chinese version, was as follows: conceptual definition or equivalence, forward-translation, back-translation, expert review, cognitive interviewing, and proofreading. In the first phase, staff and researchers working in palliative care identified conceptual definitions and equivalence of key concepts. Next, forward-translation was conducted by two researchers independently from English to Chinese. A third person facilitated discussions to produce a preliminary Chinese version. This preliminary version was then back-translated by another two researchers independently from Chinese to English. In the fourth phase, review was performed by an expert panel comprising two palliative care doctors, one palliative care nurse, one medical social worker and one health outcomes researcher, in consultation with the original developers of IPOS to refine the phrasing of each question. Subsequently, 12 English-speaking and 12 Chinese-speaking patients with advanced solid tumours completed cognitive interviewing via semi-structured interviews. Feedback on the clarity of questions was obtained from the participants and used to revise question phrasing, to ensure that items could be clearly understood within Singapore’s context of both languages. The finalised versions were reviewed by the researchers and developers of IPOS.

#### Validation

Inpatients were recruited from Singapore General Hospital; community patients were recruited from National Cancer Centre clinics, Dover Park Hospice and Assisi Day Hospice. The inclusion criteria were: i) at least 21 years old, ii) diagnosed with advanced cancer, defined as stage 4 solid tumour, iii) able to communicate in either English or Chinese, iv) aware of their advanced cancer diagnosis (assessed by the primary doctor in charge of their clinical care) and v) able to give informed consent. There were no event-based or other criteria such as hospital admission or time from diagnosis. Healthcare staff managing the patients who consented to participate were recruited for evaluation of the staff version of IPOS in English. Written informed consent was obtained for all participants. This study was approved by the Singhealth Centralised Institutional Review Board (CIRB) (Reference number 2018/2086) and all methods were carried out in accordance with relevant guidelines and regulations.

Data were collected at 2 timepoints – baseline and follow-up at 2 to 5 days later for inpatients or 7 to 21 days for community patients. At baseline, patients completed the patient version of IPOS, Functional Assessment of Cancer Therapy – General (FACT-G) and Edmonton Symptom Assessment System - revised (ESAS-r) [14, 15]. At the follow-up timepoint, patients completed the patient version of IPOS and answered a question on whether they felt their main problems or concerns had changed since baseline (Global change question). Patients chose either the English or Chinese version of IPOS and used the same version for both timepoints. At both timepoints, a staff member (either nurse or doctor) involved in the patient’s care completed the staff version of IPOS, and reported the time needed to complete the questionnaire as well as the perceived utility of the questionnaire in patient management. All staff used the English staff version of IPOS.

### Measurement tools

#### IPOS

A 17-item questionnaire comprising 3 subscales: Physical Symptoms subscale (10 items), Emotional Symptoms subscale (4 items) and Communication and Practical Issues subscale (3 items) [4]. Each item was scored on a 5-point Likert-type scale from 0 (best) to 4 (worst) for each individual item for patients. Staff versions included the same questions with an additional option of “cannot assess”. In addition, responses could be marked as “not applicable” or “don’t want to tell”. Total and subscale scores were summed, with higher scores indicating poorer outcomes.

#### Functional assessment of Cancer therapy – general (FACT-G)

A 27-item quality of life measure that comprises four subscales of Physical Wellbeing, Social Wellbeing, Emotional Wellbeing and Functional Wellbeing [14]. Each item was measured on a 5-point Likert Type scale. Higher scores indicate better functioning. This was used to assess construct validity.

#### Edmonton symptom assessment system-revised (ESAS-r)

A 9-item measure with visual analogue scales (scored from 0 to 10) for pain, shortness of breath, nausea, depression, activity, anxiety, wellbeing, drowsiness, and appetite [15]. Higher total summed scores indicate high symptom burden. This was also used to assess construct validity.

#### Global change question

At follow-up, patients were asked *“Since the questionnaire was last completed, thinking about your main problems and concerns, would you say that: things have got much better, things have got a little better, there has been no change, things have got a little worse, or things have got much worse”*. This was used to determine the patients included in the sample to assess test-retest reliability.

#### Staff feedback

Staff were asked to report the amount of time taken to complete the questionnaire on a 3-point scale (< 5 min, 5–10 min, > 10 min), and their opinion on the relevance of the staff IPOS for assessing patient outcomes on a 4-point Likert scale (very relevant, slightly relevant, slightly irrelevant, and very irrelevant).

Demographic and clinical data were collected from medical records. English and Chinese versions of patient responses were analysed separately.

### Descriptive statistics

For total and subscale scores, floor and ceiling effects were calculated, and a threshold of 15% was deemed acceptable [16].

### Validity

To determine structural validity, we conducted a confirmatory factor analysis (CFA) for patient and staff IPOS responses at baseline using the pre-determined subscales of IPOS [5]. Responses were treated as ordered categorical data. We hypothesized that using the three pre-determined subscales of IPOS (Physical Symptoms subscale, Emotional Symptoms subscale, Communication and Practical Issues subscale) with each item loaded onto one subscale, the goodness-of-fit indices would be within acceptable limits (Comparative fit index [CFI] and Tucker-Lewis-Index [TLI] of more than 0.90 and Root Mean Square Error of Approximation [RMSEA] of less than 0.08) [17].

Known-group validity was evaluated using Student’s t-test comparing total and subscales between patient responses obtained in the inpatient vs community settings at baseline. We hypothesized that inpatients would be more unwell and have more problems and concerns than community patients. Therefore, we anticipate that inpatients would have higher IPOS scores than community patients.

Construct validity was tested by correlating IPOS subscales with the respective total and subscale scores of ESAS-r and FACT-G, using Pearson’s correlation coefficients (r) and data from baseline. We hypothesized that there were correlations of r ≥ |0.3| between the following scores, as they measure similar themes as found in previous studies [5]:

- IPOS Physical Symptoms subscale vs patient ESAS-r Total and FACT-G Physical Wellbeing subscale
- IPOS Emotional Symptoms subscale vs patient ESAS-r Total and FACT-G Emotional Wellbeing subscale
- IPOS Communication and Practical Issues subscale vs FACT-G Social Wellbeing subscale

### Reliability

Internal consistency was evaluated by calculating Cronbach’s alpha for the total and subscales using staff and patient responses at baseline. Following the original validation study, we adopted a lower threshold (0.60 instead of 0.80 normally accepted). Due to the non-redundant nature of IPOS, we expected less agreement between individual questions in each subscale as each question assessed for a different aspect of QOL [5].

Inter-rater reliability between patient and staff IPOS was tested by calculating the intraclass correlation coefficient (ICC) using an analysis of variance (ANOVA) estimator for total and subscale scores.

For patients who responded “no change” to the Global change question, patient IPOS scores at baseline and follow-up were used to evaluate for test-retest reliability. This was done by calculating the ICC using an ANOVA estimator for total and subscale scores.

### Healthcare worker’s acceptability

Proportions were calculated for staff responses on the amount of time spent to complete the questionnaire and their opinion on the relevance of the staff IPOS for assessing patient outcomes.

### Sample size

To establish construct validity, a sample size of 113 per language was needed to detect a Pearson’s correlation coefficient of at least 0.3 between the summative symptom assessment score in IPOS and ESAS-r, with 90% power at 5% two-sided type 1 error.

### Missing data

IPOS and ESAS-r responses with multiple answers for a single item, missing responses, “not applicable” response, “don’t want to tell” response or “cannot assess” response for any of the 17-item closed-ended questions were removed from analysis [18]. In cases with missing or “not applicable” responses for FACT-G, values were replaced with the mean of their respective subscales, with all the subscales being at least 50% completed [19].

### Analysis

The R lavaan package was used for CFA. All other analyses were done using STATA 12.0.

## Results

### Subject characteristics

**Fig. 1:**
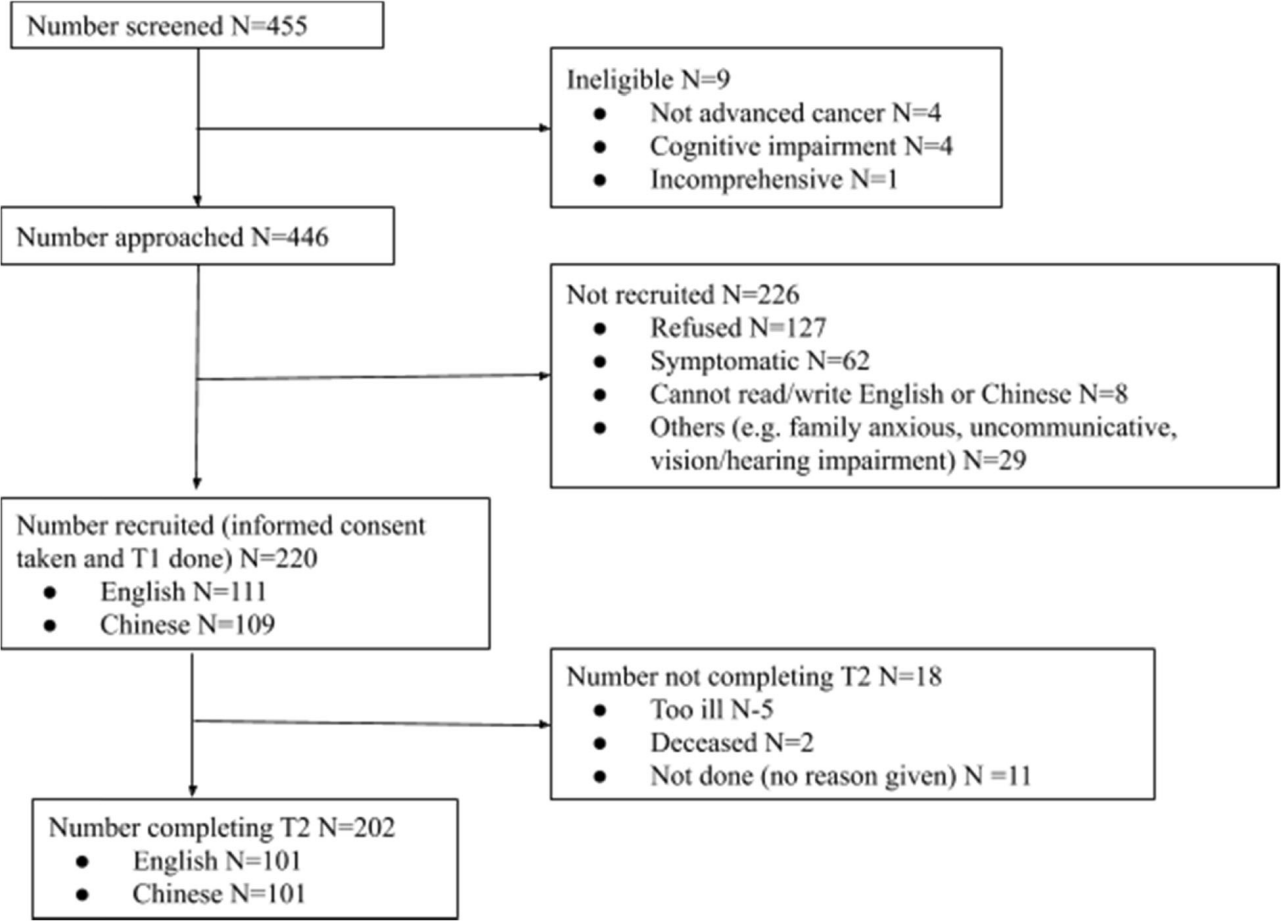
Flowchart for patient recruitment

Patients and staff were recruited from July 2018 to November 2019. Figure 1 shows patient recruitment. A total of 111 and 109 patients completed the English and Chinese questionnaires respectively at baseline. At follow-up, 101 patients completed the questionnaire for each language. For staff versions, 86 and 79 participants who answered the English and Chinese questionnaires respectively had a staff member in their healthcare team respond at baseline; 70 English questionnaire responders and 62 Chinese questionnaire responders had a staff IPOS response at follow-up (Table 1).

**Table 1:** Patient demographics and clinical characteristics

Characteristics	English (***N*** = 111)		Chinese (***N*** = 109)	
	N	%	N	%
**Age, Mean (SD)**	59.8 (12.4)		62.9 (11.2)	
**Gender**				
Men	55	49.5	53	48.6
Women	56	50.5	56	51.4
**Ethnicity**				
Chinese	72	64.9	109	100
Malay	27	24.3	0	0.0
Indian	8	7.2	0	0.0
Other	4	3.6	0	0.0
**Marital status**				
Married	83	74.8	76	69.7
Single	17	15.3	21	19.3
Divorced	6	5.4	4	3.7
Widowed	5	4.5	8	7.3
**Education**				
No formal education	3	2.7	9	8.3
Primary school	13	11.7	42	38.5
Secondary school	55	49.6	48	44.0
Post-secondary level	40	36.0	9	8.3
Missing	0	0.0	1	0.9
**Primary cancer diagnosis**				
Digestive organs – colorectal	33	29.7	40	36.7
Digestive organs – non-colorectal	22	19.8	15	13.8
Lung	18	16.2	25	22.9
Genitourinary tract	13	11.7	13	11.9
Breast	14	12.6	10	9.2
Other cancers	10	9.0	6	5.5
Unknown/ missing	1	1.0	0	0.0
**Mode of administration (baseline)**				
Interviewer-administered	38	34.2	57	52.3
Self-completed	63	56.8	48	44.0
Self-completed with help from others	10	9.0	4	3.7
**Setting (baseline)**				
Inpatient	63	56.8	59	54.1
Outpatient	48	43.2	50	45.9

### Descriptive statistics, distribution, and missing values

There were no floor or ceiling effects observed for the total IPOS scores, IPOS Physical Symptoms subscale and IPOS Emotional Symptoms subscale at baseline (Table 2). Floor effects were seen for IPOS Communication and Practical Issues subscale. Specifically, 16.4 and 18.5% of patients completing the English and Chinese versions respectively had the lowest (worst) possible scores for the Communication and Practical Issues subscale.

**Table 2:** Percentage of patients who reported the lowest and highest possible score, intraclass correlation coefficients for test-retest reliability for patients reporting no global change and Cronbach’s alpha for patient and staff responses at baseline

	Number of items	Mean (SD)	Descriptive statistics		Test-retest		Cronbach’s alpha	
			% with min score	% with max score	ICC	95% CI	Patient	Staff
**English**		*N* = 111	*N* = 111		*N* = 30		*N* = 111	*N* = 143
**Total**	17	17.77 (10.99)	1.80	0.00	0.80	0.67–0.93	0.88	0.84
Physical	10	8.50 (6.95)	9.01	0.00	0.77	0.63–0.92	0.84	0.74
Emotional	4	5.85 (3.96)	7.21	1.8	0.72	0.55–0.90	0.80	0.82
Communication and Practical Issues	3	3.16 (2.31)	16.21	0.00	0.53	0.27–0.79	0.58	0.65
**Chinese**		*N* = 109	*N* = 109		*N* = 27		*N* = 109	–
**Total**	17	14.45 (10.10)	1.83	0.00	0.88	0.80–0.97	0.84	–
Physical	10	7.87 (7.19)	13.76	0.00	0.93	0.87–0.98	0.82	–
Emotional	4	4.52 (3.30)	11.01	0.00	0.80	0.66–0.94	0.68	–
Communication and Practical Issues	3	2.87 (2.19)	18.34	0.00	0.45	0.15–0.76	0.29	–

### Validity

From the confirmatory factor analysis for structural validity, the three-factor model based on the existing factor structure (Physical Symptoms, Emotional Symptoms, Communication and Practical Issues) was a good fit with CFI and TLI greater than 0.9 and RMSEA less than 0.08. For the patient English version, CFI, TLI and RMSEA were 0.986, 0.983 and 0.063 respectively. For the patient Chinese version, CFI, TLI and RMSEA were 0.972, 0.968 and 0.064 respectively. Lastly, for the staff version in English, CFI, TLI and RMSEA were 0.971, 0.966 and 0.082 respectively.

At baseline, as hypothesized, patients in the inpatient setting had higher total and subscale scores than community patients (Table 3), supporting known-group validity. For the English version, mean total scores were 21.1 for inpatients and 13.3 for community patients (*p* < 0.01); for the Chinese version, mean total scores were 17.6 for inpatients and 10.7 for community patients (*p* < 0.01).

**Table 3:** Difference between IPOS total and subscale scores for inpatient vs community at baseline

	Mean (SD)		Difference in means	***p***
	Inpatient	Community		
**English**	*N* = 63	*N* = 48		
**Total**	21.07 (10.43)	13.34 (10.21)	7.80	< 0.01
Physical	10.76 (6.63)	6.09 (6.67)	4.61	< 0.01
Social	6.84 (4.03)	4.51 (3.48)	2.33	< 0.01
Communication and Practical Issues	3.48 (2.38)	2.74 (2.11)	0.74	0.05
**Chinese**	*N* = 59	*N* = 50		
**Total**	17.57 (10.40)	10.69 (8.36)	6.90	< 0.01
Physical	9.14 (7.15)	4.57 (5.45)	4.57	< 0.01
Social	5.08 (3.28)	3.84 (3.23)	1.24	0.02
Communication and Practical Issues	3.36 (2.26)	2.29 (1.97)	1.07	< 0.01

There were significant correlations between the physical subscales of IPOS and FACT-G (English: *r* = − 0.731, *p* < 0.01; Chinese: *r* = − 0.786, *p* < 0.01). Similarly, the emotional symptom subscales of IPOS and FACT-G showed significant correlations (English: *r* = − 0.664, *p* < 0.01; Chinese: *r* = − 0.608, *p* < 0.01). Moreover, there were good correlations between the total ESAS-r scores and both the IPOS Physical Symptoms subscale (English: *r* = 0.750, *p* < 0.01; Chinese: *r* = 0.708, *p* < 0.01) and the IPOS Emotional Symptoms subscale (English: *r* = 0.632, *p* < 0.01; Chinese: *r* = 0.614, *p* < 0.01). However, the results did not show strong correlations between the IPOS Communication and Practical Issues subscale and the FACT-G Social Wellbeing subscale (English: *r* = − 0.142, *p* = 0.138; Chinese: *r* = − 0.368, *p* < 0.01) (Table 4).

**Table 4:** Correlation between IPOS and FACT-G subscales and ESAS-r total

	IPOS Total	IPOS Physical Symptoms	IPOS Emotional Symptoms	IPOS Communication and Practical Issues
	r			
**English (*N*** **= 111)**				
**FACT-G Total**	**−0.71**	**−0.62**	**−0.60**	**−0.44**
Physical	**−0.79**	**−0.73**	**−0.66**	**− 0.41**
Social	− 0.06	− 0.04	− 0.02	− 0.14
Emotional	**− 0.62**	**− 0.47**	**− 0.66**	**− 0.40**
Functional	**− 0.54**	**− 0.50**	**− 0.40**	**− 0.37**
**ESAS-r Total**	**0.77**	**0.75**	**0.63**	**0.34**
**Chinese (*N*** **= 109)**				
**FACT-G Total**	**−0.73**	**−0.64**	**− 0.67**	**− 0.36**
Physical	**− 0.80**	**− 0.78**	**−0.60**	**− 0.36**
Social	−0.07	0.07	−0.13	**− 0.36**
Emotional	**−0.54**	**− 0.48**	**−0.60**	− 0.07
Functional	**−0.52**	**− 0.47**	**−0.47**	− 0.21
**ESAS-r Total**	**0.75**	**0.70**	**0.61**	**0.35**

Values in bold indicate *p* < 0.01

### Reliability

The total IPOS scores, Physical Symptoms subscale, and Emotional Symptom subscale scores showed good internal consistency for all versions (Table 2). For total IPOS scores, Cronbach’s alpha was above 0.80 for the patient English, patient Chinese, and staff versions. Similarly, Cronbach’s alpha was above 0.80 for IPOS Physical and Emotional Symptoms subscales in the patient English, patient Chinese, and staff versions. Notably, for the IPOS Communication and Practical Issues subscale, Cronbach’s alpha was 0.65 for the staff version, but lower than the threshold of 0.6 for both patient versions (English = 0.58 and Chinese = 0.29).

There was poor inter-rater reliability for both languages, with ICC ranging from 0.00–0.43 between patients and staff responses at both time points (Table 5).

**Table 5:** Inter-rater agreement between patient reported IPOS and staff reported IPOS for inpatients

	ICC	95% CI	ICC	95% CI
**English**	Baseline (*N* = 76)		Follow-up (*N* = 31)	
**Total**	0.21	0.00–0.42	0.38	0.08–0.68
Physical	0.15	0.00–0.37	0.35	0.03–0.66
Emotional	0.21	0.00–0.42	0.31	0.00–0.63
Communication and Practical Issues	0.16	0.00–0.38	0.23	0.00–0.56
**Chinese**	Baseline (*N* = 67)		Follow-up (*N* = 31)	
**Total**	0.43	0.24–0.63	0.00	0.00–0.35
Physical	0.26	0.00–0.48	0.02	0.00–0.38
Emotional	0.42	0.23–0.62	0.04	0.00–0.39
Communication and Practical Issues	0.26	0.04–0.49	0.00	0.00–0.35

A total of 30 English-speaking and 27 Chinese-speaking patients responded “no change” for the Global change question at follow-up and were used to assess test-retest reliability (Table 2). For English-speaking patients, the mean durations between baseline and follow-up were 2.68 (SD = 0.891) days for inpatients and 8.38 (SD = 3.66) days for community patients. We observed similar durations for the Chinese-speaking patients, with the mean duration between baseline and follow-up at 2.62 (SD = 0.93) days and 8.39 (SD = 5.82) days for community patients. There was good test-retest reliability for total IPOS scores for both languages (ICC = 0.80 for English and ICC = 0.88 for Chinese). For the English version, test-retest reliability was moderate for the IPOS Physical Symptoms subscale (ICC = 0.77) and the IPOS Emotional Symptoms subscale (ICC = 0.72), but low for the Communication and Practical Issues subscale (ICC = 0.53). For the Chinese version, test-retest reliability was good for the IPOS Physical Symptoms subscale (ICC = 0.93) and the IPOS Emotional Symptoms subscale (ICC = 0.80) but low for the Communication and Practical Issues subscale (ICC = 0.45).

The mean (95% confidence interval) of the changes for IPOS Total among the remaining participants who have an improvement and a deterioration were 0.78 (− 1.6 to 3.2) and 2.7 (− 1.9 to 7.5) respectively for English responses and 1.2 (− 0.9 to 3.4) and 5.5 (− 6.2 to 17.2) respectively for Chinese responses.

### Healthcare worker’s acceptability

In total, 97.5% of staff completed the IPOS staff form in less than 5 min, 2.5% taking between 5 and 10 min, and none taking more than 10 min. 54.7% found the IPOS staff tool “very relevant” and an additional 42.2% found it “slightly relevant” to assessing the outcomes of their patients, with the remaining 3.1% finding the tool “slightly irrelevant” or “very irrelevant”.

## Discussion

The English and Chinese versions of patient-reported IPOS were shown to be a valid and reliable PROM overall for palliative care outcomes among advanced cancer patients in Singapore. The three-factor model was confirmed, with good known-group validity between inpatients and community patients. Furthermore, the patient versions showed good construct validity with ESAS-r and FACT-G, except for the Communication and Practical Issues subscale. Both patient and staff-reported IPOS also showed good internal consistency, with the exception of the IPOS Communication and Practical Issues subscale. Test-retest reliability was found for both language versions. However, the results showed poor inter-rater reliability between patient and staff reported scores.

Overall, there was good construct validity between the total and subscale scores of IPOS, and the FACT-G domains scores. Surprisingly, there was a strong correlation between the IPOS Emotional Symptoms subscale and FACT-G Physical Wellbeing subscale (English: *r* = − 0.668; Chinese: *r* = − 0.601). This might be because FACT-G Physical Wellbeing subscale includes items about the emotional effects of physical problems, such as “I am bothered by the side effects of treatment”, which is similar to items in the IPOS Emotional Symptoms subscale, such as “Have you been feeling anxious or worried about your illness or treatment?” The IPOS Communication and Practical Issues subscale had poor correlation with all FACT-G and ESAS-r scores, echoing findings in other IPOS validation studies [5, 8, 9]. The consistently poor correlation between this subscale and other PROMs suggests that the types of problems in this Communication and Practical Issues subscale are not measured by existing PROMs.

Our study population comprised of patients with advanced cancer regardless of whether they were known to any palliative care services, setting it apart from other validation studies of IPOS in patients receiving palliative care [5, 8, 10]. That IPOS showed overall good measurement properties in both English and Chinese with good construct validity, internal consistency and test-retest reliability suggests that IPOS could be used as a screening tool to identify patients with problems or concerns that could trigger a referral to the specialist palliative care team [20]. The IPOS tool could also be used to characterise the profile of palliative care concerns in the wider population of patients with advanced cancer, regardless of whether they are referred to palliative care services or not [21]. The availability of other language versions of IPOS may facilitate comparison of palliative care outcomes across various language contexts in different countries as well as allow for international studies of palliative care outcomes involving patients from different countries or cultural contexts [5, 8–10].

There was poor patient-staff agreement in both languages when used in this study population, unlike other studies [5, 8]. This could be because majority of the staff included in this study were ward nurses in the acute hospital setting, who may be less attuned to the patient’s problems and concerns compared to palliative care clinicians surveyed in the other validation studies. For use in this context, where giving training to acute ward nurses to assess palliative care problems and concerns may not be feasible, family caregivers could be explored as alternative proxy respondents who could potentially have greater agreement with patient-reported scores compared to healthcare proxies [22, 23]. As patients at more advanced stages of disease may have reduced physical and mental capacity to self-respond to such questionnaires, it is worthwhile to further explore how the proxy measure can be administered more reliably in the local context.

This study has several limitations. Firstly, stable patients were identified using the Global change question to assess test-retest reliability. This method of identifying stable patients is limited as patients’ main problems or concerns might have remained the same but have experienced changes in other unrelated aspects of their health that were also captured by IPOS (e.g. communication). In particular, this was seen in the Japanese validation study, where questions in the IPOS Communication and Practical Issues subscale had lower test-retest reliability than other subscale questions^10^. Secondly, the study did not assess for responsiveness as the time frame between baseline and follow-up was too short for patients’ conditions to have changed appreciably. Therefore, IPOS’s utility to track patient outcomes over time at an individual level, or to compare various palliative care interventions in research studies longitudinally between populations remains to be determined in the context of advanced cancer patients in Singapore. Lastly, this study only involved advanced cancer patients, limiting the use of the patient IPOS in patients with other conditions, including early-stage cancer. Thus, future studies may expand the study population to show the generalizability of IPOS’s utility.

## Conclusion

Overall, we found the patient IPOS tool to be valid and reliable in both English and Chinese when used in advanced cancer patients in Singapore. IPOS in both languages demonstrated good structural validity, known group differences, good construct validity, good internal consistency, and demonstrated test-retest reliability, with the exception of the Communication and Practical Issues subscale. The patient versions can be used as a brief global outcome measure of palliative care problems and concerns in this population studied. The proxy staff versions showed poor inter-rater reliability with the patient reported versions. Thus, further research needs to be done to develop proxies to assess patient outcomes.

## Data Availability

The datasets used and analysed during the current study are available from the corresponding author on reasonable request.

## Abbreviations

IPOS: Integrated palliative care outcome scale
ESAS-r: Edmonton symptom assessment system-revised
FACT-G: Functional assessment of cancer therapy–general
ICC: Intra-class correlation coefficient
PROM: Patient reported outcome measures
POS: Palliative care outcome scale
QOL: Quality of life
CIRB: Centralised institutional review board
CFA: Confirmatory factor analysis
CFI: Comparative fit index
TLI: Tucker-lewis-index
RMSEA: Root mean square error of approximation
ANOVA: Analysis of variance
